# This issue in *BJUI Compass*: March 2020

**DOI:** 10.1002/bco2.10

**Published:** 2020-03-27

**Authors:** John W. Davis

**Figure:**
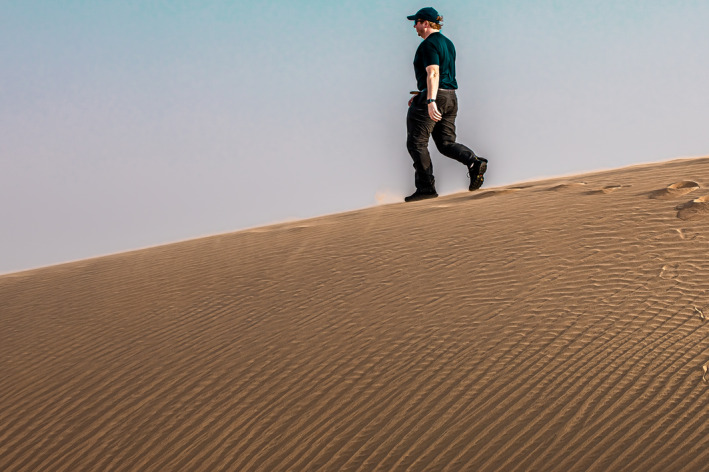
*A desert walk in Abu Dhabi … your editor is wandering the globe seeking high quality papers for* BJUI Compass

Welcome to the inaugural issues of the *BJUI Compass—*the latest expansion in peer reviewed publishing from the BJU International family of publications. *BJUI* has been published since 1929 and is soon to wrap up a highly successful eight‐year run under the leadership of Editor Prokar Dasgupta. It has been a privilege to serve this high‐energy team as it has been at the forefront of pushing academic publishing further into the online/digital era.

*BJUI* is the flagship publication of the organization and it seeks to find and publish the highest‐impact scholarly articles from all specialties of urology. *BJUI Compass* will be an important companion piece to the academic and clinical care communities, as it will emphasize sound science principles from its articles, and break new ground into the rapidly expanding area of open access publishing, with the final product will be available to everyone with internet access.

The BJU International Trustees came up with the name *BJUI Compass* for this new journal. It is a name I really like*—*as a frequent traveler for academic events throughout the world*—*the theme of direction and travel is fitting for our specialty. Furthermore, urology covers a lot of ground in the various subspecialties*—*oncology, andrology, functional, pediatrics, basic science, etc. Therefore, we have chosen an editorial board with a broad range of specialty backgrounds, and we have chosen a simple four‐directional theme for our articles: reviews, academics, clinical utility, and surgical innovation. In “compass” terms, we would equate these to directional movement: To the literature, To the drawing board, To the clinics, and To the future.

In this issue of *BJUI Compass* …

1. *To the literature*. For our first review article, Pessoa et al tackle the recent trends in enhanced recovery after urologic surgery. Of course many pioneering surgeons have made significant improvements in patient recovery and outcomes through minimally invasive surgery procedures including laparoscopy and robotics. However, the comprehensive perioperative care of the operation was less often seen as an opportunity for research and innovation until well after my formal training. The home base for "Eras" was radical cystectomy but, as the article shows, the principles have been transferred into many other urologic procedures. It may seem like a minor advance, but when I was a resident, cystectomy patients had a nasogastric tube for days at a time*—*until clear return of bowel function. Now, we do not even place one. Now multiply that change over the course of counseling, optimization, carb loading, no bowel prep, VTE prophylaxis, pain control, early mobilization, etc…
2. *To the drawing board*. Klocker et al present a novel multivariate risk score for prostate cancer detection. Of course, this is one of the most published topics in urology*—*diagnosing prostate cancer. These authors use a new prediction model to improve the specificity of screening*—*above that seen with percent free Prostate Specific Antigen. The take home statistic stands for itself: at 90% selected sensitivity, it yields 43% specificity, 95% negative predictive value, and positive predictive value of 25%. A marker panel like this may be a useful complement to clinical judgment and/or MRI for optimized biopsy selection.
3. *To the clinic*. Das et al report on a common clinical dilemma*—*how to make appropriate selections for pharmacologic therapy when there is a lack of head to head comparison data, and less reported post clinical trial release. Specific to the National Health Service (UK) and perhaps other systems, clinicians must either select abiraterone for castrate resistant prostate cancer or enzalutamide, but cannot use both or cross over routinely. In the authors’ comparison, enzalutamide had a more favorable biochemical progression result, but no difference in overall survival or radiologic progression. Their design is only hypothesis generating but would suggest that clinicians can go with either drug, personal comfort with administration/side effects, or perhaps decide if the differences in fatigue vs hypertension are worthy of differentiation.
4. *To the future*. Adamic et al present a novel technique from one of the leading robotic surgery centers in pediatric urology: the Mitrofanoff (ileocystoplasty) with robotic technique. This is a moderate size cohort series that shows the step‐by‐step technical points and cohort outcomes. Of course this is a heterogeneous population to apply a new technique, but the authors make their case for procedure safety and feasibility. Of note, while the radical prostatectomy is the high‐volume anchor procedure for most robotics programs, the overall field of reconstructive urology is a major area of growth in robotics and adult surgeons should also study these advances in pediatric robotic construction to maximize their potential for offering minimally invasive reconstruction.

